# Teamwork skills in higher education: is university training contributing to their mastery?

**DOI:** 10.1186/s41155-022-00207-1

**Published:** 2022-02-10

**Authors:** Elena De Prada, Mercedes Mareque, Margarita Pino-Juste

**Affiliations:** 1 Department of English, French and German, Faculty of Business and Tourism, University of Vigo, Campus Universitario, 32004 Ourense, Spain; 2 ECOBAS (Economics and Business Administration for Society), Department of Financial Economics and Accountancy, Faculty of Business and Tourism, University of Vigo, Campus Universitario, 32004 Ourense, Spain; 3 Department of Didactics, School Organization and Research Methods, Faculty of Education Sciences and Sports, University of Vigo, Campus A Xunqueira, 36005 Pontevedra, Spain

**Keywords:** Teamwork skills, Gender, Academic year, GPA, Higher education

## Abstract

Teamwork skills are considered essential for personal, academic and professional achievement, so universities are increasingly integrating them into their syllabuses. However, little is known about how some specific features of students and their educational development can affect their acquisition. Accordingly, this study aims to fill this gap and describe higher education students’ mastery of teamwork skills and its relation to certain socio-academic variables (gender, academic year and grade point average—GPA). With the aim of determining the level of teamwork skills among university students, an observational, transversal descriptive study was designed with an intentional sample of Spanish university students. The sample is made up of 615 social science degree students. The results suggest significant gender differences, highlighting that female students outdid their male counterparts in most teamwork skills, except leadership. Likewise, students’ skills improved as they progressed in their studies, particularly those skills related to adaptability and decision-making. Finally, a positive relationship was observed between teamwork skills and GPA, except for interpersonal development. A regression analysis confirmed the influence of both academic year and GPA for women whilst no effect was detected in the case of men. Based on these results, it is suggested to make changes in university education programmes to compensate for the influence of socio-academic factors and benefit from the most positive features of each gender regarding teamwork to achieve an equal and fair higher education.

## Introduction

Today most higher education institutions highlight the necessity of including both hard and soft skills in their syllabus to meet the required personal, academic and professional demands for a successful career. Hard skills are considered to be the technical knowledge and experiences needed to carry out a job, whilst soft skills are interpersonal qualities, also understood as an individual’s set of social skills and personal attributes. Therefore, soft skills refer to a broad group of skills, behaviours, and personal qualities that enable individuals to function efficiently in their environment, have effective relationships, carry out their work professionally, and achieve the goals they are set (Lippman et al., 2014). Soft skills are considered excellent complements to traditional hard skills at university due to their significant role in the current context. However, although soft skills are considered important as hard skills, there is a lack of consensus regarding their characterisation and implementation (Yan et al., 2019).

Universities are aware that having an adequate level of education and training not only implies a certain mastery of the contents of a given syllabus; students also need to fully develop the necessary skills to access the job market (García, 2016). According to Robles (2012), in fact, employers consider social skills to be a significantly important attribute for job seekers, demanding that their new employees’ soft skills are as well consolidated as their hard skills, given that current job positions require additional qualities that were previously not demanded (Pitan, 2017). Thus, both employers and academic institutions are slowly becoming aware of the need to equip individuals with the competencies, skills and knowledge that will not only facilitate their incorporation into the job market after their studies but also support their professional development throughout their career so that they can successfully adapt to changes in the job market.

Within this context, teamwork skills have been gaining close attention, as they are considered essential competencies in an increasingly more globalised, dynamic and complex world. New employees are asked if they have teamwork skills, can resolve specific work issues or have the required skills to handle the new challenges posed by today’s society (Baneres & Conesa, 2017). Universities are not oblivious to society’s needs, specifically to the demand of companies for students and future workers to be trained in soft skills. Over the past few years, universities have manifested their concern with students’ mastery of soft skills, specifically those related to learning to work in teams, given their correlation with employability. Recruiters are looking for employees with soft skills, as they are aware of the link between the former and the successful maintenance and execution of a job (Blaszczynski & Green, 2012). Therefore, in the present day, job adverts frequently list soft skills—including teamwork—as a requirement (Clares et al., 2019).

Little research focuses on students’ acquisition level of the different teamwork skills, which will aid ongoing learning throughout their careers. The same happens with research focused on establishing the relationship between teamwork skills and students’ specific socio-academic features. In this sense, studies such as the ones carried out by Al-Alawneh and Ashour (2011), Al-Alawneh et al. (2011), Beigi and Shirmohammadi (2012), Chamorro-Premuzic et al. (2010), Chapman and Van Auken (2001), Ilias et al. (2012), Lozano-Rodríguez et al. (2020), Park et al. (2015) and Rodríguez-Gómez et al. (2018) have tried to establish the possible relationships between teamwork skills and gender, academic experience or academic performance.

The objective of the present study is to describe higher education students’ mastery level of teamwork skills and its relation to certain socio-academic variables, to introduce changes in university training programmes that can respond to the demands of companies and strengthen their employability. In order to carry out this objective, an observational, transversal descriptive study was devised and tested with an intentional sample of Spanish university students, using the Teamwork Skills Questionnaire (TSQ) (O’Neill et al., 1999), which measures an individual’s skill level to work efficiently in a team. This research contributes to the existing literature offering new empirical evidence about teamwork skills levels in Spanish university students on the one hand and adding new insights about the influence of gender and academic factors on teamwork skills on the other.

## Background

### Soft skills for teamwork

Soft skills are considered essential elements of employees’ development. The acquisition of these skills enables attitudinal and behavioural change in workers, as well as increased productivity and well-being (Sitthisomjin et al., 2014). Soft skills facilitate effective teamwork, which is an integral part of the execution of many professions (Vaughan et al., 2019).

Therefore, students in both compulsory and further education need to be trained in these skills if they are to become graduates capable of competing in the variety of situations they may come up against in the workplace (Ali et al., 2017). Following this line of research, several works have tried to identify the most relevant soft skills that the job market demands from the point of view of students and company supervisors. Durán-Aponte and Durán-García (2012) highlight the relevance of ethical commitment, personal skills, teamwork and professional responsibility. Clemente-Ricolfe and Escribá-Pérez (2013) include analysis capacity, problem solving and teamwork. Freire et al. (2011) confirm that the most valued skills in the job market are responsibility, learning capacity, motivation, concern for quality and teamwork. Accordingly, we can observe that the common denominator of this research is that teamwork is one of the key soft skills that students have to acquire for their future professional success.

Furthermore, soft skills may contribute to their success in many academic and personal situations. The evidence has demonstrated that soft skills promote a series of tangible benefits for health, welfare, relationships, education and work. Given the variety of soft skills, we have turned to the systematic reviews in this area to determine which are the most frequently cited in education and the professional sphere (Gates et al., 2016; Lippman et al., 2014). Thus, we have observed that the highest valued soft skills by employers are integrity, communication, courtesy, responsibility, social skills, positive attitude, professionalism, flexibility, teamwork, creativity and work ethic.

It is worth pointing out the varying approaches to the definition of teamwork. Thus, in line with Hare (2010), our study interprets teamwork as a group of individuals with (a) commonality of goals across members, (b) synergy that emerges from members’ interdependence and (c) size, with at least two members viewed as sufficient. In addition, we should bear in mind that “teamwork” is sometimes considered a skill in itself; in this study, however, our focus is on measuring the soft skills that enable good teamwork, along similar lines as Bonavia et al. (2015).

The literature has considered many techniques and measurements for evaluating teamwork skills (Bonavia et al., 2015). For this study, we have chosen to use the test designed by O’Neill et al. (1999), a multi-dimensional scale analysing various dimensions of perceptions of teamwork. It includes the measurement of six key soft skills for teamwork: coordination, decision-making, leadership, interpersonal development, adaptability, and communication.

Finally, it is essential to note that studies designed to observe teamwork skills should consider the country’s social and cultural context since socio-cultural features can determine individuals’ behaviour and attitude towards teamwork. For example, one of the dimensions of culture, collectivism versus individualism, has been demonstrated to influence teamwork since the two positions will approach group work in a different way (Galanes et al., 2004).

### Teamwork skills and socio-academic factors

Previous studies have identified a relationship between student attitudes towards teamwork skills and specific student characteristics such as gender, academic experience or academic performance. However, it is important to mention that research is scarce in the last two variables mentioned. It should also be noted that there is some controversy regarding the results of the studies consulted, especially concerning the academic experience and academic performance, probably due to the diverse cultural and educational contexts and the different measures used, as previously stated.

In what follows, we will describe relevant research about the three variables under consideration, including the context, instrument and data used.

### Gender

Regarding the possible influence of gender on teamwork skills, it is essential to consider that male and female differences are present in all societies in many spheres (Ellemers, 2018), with degrees of variation created by various cultural influences. Economic-social development and religion, among others, can determine variations in gender (Best & Puzio, 2001). For this reason, providing information about participants and the contexts where studies are conducted is fundamental to interpret the results correctly and advance in the field.

Rodríguez-Gómez et al. (2018) analyse ten essential competencies in Spanish university students, including teamwork. They found significant differences in six of these competencies, indicating that women obtain a higher average score than men in five of the six competencies, teamwork being one of them. They emphasise the greater degree of statistical significance in the difference of means in teamwork competence. Al-Alawneh et al. (2011) investigate whether there are statistically significant differences in teamwork skills ratings in Jordan university students. For this purpose, they analysed six competencies related to teamwork (coordination, decision-making, leadership, interpersonal development, adaptability, and communication) and reported significant differences in communication skills and interpersonal development, the latter showing a higher average score in the case of women. Other studies also highlight gender differences in specific teamwork skills, although they do not clarify if those differences favour men or women. In this sense, Ilias et al. (2012), in a Malaysian context, or Al-Alawneh and Ashour (2011) for graduates of career and technical education institutions in Jordan, study the same six teamwork skills as Al-Alawneh et al. (2011). The former revealed significant differences between genders regarding adaptability and leadership, whilst the latter found significant differences in coordination, communication and interpersonal development.

### Academic experience

Academic experience has been positively related to teamwork skills development, emphasising that final-year students are more likely to possess the required competencies for teamwork (Burdett & Hastie, 2009). However, as previously indicated, the research conducted in diverse contexts using different measures presents some inconsistencies in its findings. The related literature generally uses two indicators, the students’ age or the academic year. In our study, we have used the second indicator.

Rodríguez-Gómez et al. (2018) aimed to describe students’ perception of their level of competence in ten basic competencies related to assessment, including teamwork in a Spanish university context. They pointed out significant differences in teamwork for the academic experience variable, using the academic year as an indicator. They observed a substantial increase in teamwork competence from the second year onwards. Final year students reported the highest degree of development of teamwork competence (in the last year, the mean of the competence is $\overline{x}$ = 5.24 compared to the mean of $\overline{x}$ = 4.85 in the second year). In this way, students perceived that they improved this competence at the end of their university training. However, in a different context, Jordan, Al-Alawneh et al. (2011) did not find significant differences between students’ study level and the six analysed teamwork skills.

As indicated, other researchers use the age of university students to determine the academic experience and associate it with attitudes towards teamwork. In this respect, Payne & Monk-Turner (2006) found moderate relationships between USA university students’ age and some aspects of their attitude towards teamwork skills; specifically, older students considered contributing to other group members’ learning. Something similar happens with more senior students’ willingness to take on leadership roles, as Burdett and Hastie (2009) reported. Finally, regarding favourable or unfavourable attitudes towards teamwork, Beigi and Shirmohammadi (2012) concluded that age was not relevant in an Iranian context. Concerning this cultural context, it is essential to consider that the authors highlight that Iranian organisational culture focuses on individual work rather than team collaboration and report that Iranians, compared to other nationalities, are considered less effective in teamwork activities.

### Academic performance

Academic performance is an indicator of the learning level achieved by students, and, for this reason, the education system considers it of particular relevance (Reyes, 2003). Academic performance has been defined in different ways (Alcaide, 2009), and two measures are normally used for their assessment, academic grades or objective tests (Matas, 2003). Following Cascón (2000), our study will use students’ academic performance GPA (grade point average) as the measure. This author found that the grades obtained in successive assessments and their corresponding point average are good criteria for measuring students’ academic performance. The different ways of measuring academic results must be considered to interpret research findings correctly.

In this sense, Lozano-Rodríguez et al. (2020), in Mexican universities, observed a significant correlation between teamwork skills and academic achievement, calculated using the grades obtained at the end of the academic term. Park et al. (2015) obtained similar findings in a South Korean context. They highlight that teamwork learning can improve academic performance.

Regarding students’ attitudes towards teamwork, it is important to consider that different cultural and educational contexts can organise and assess teamwork differently. Accordingly, students’ attitudes can vary depending on the perception that teamwork affects their marks (Burdett & Hastie, 2009). Likewise, it can be influenced by universities’ focus on teamwork skill training. It has been emphasised that assigning teamwork activities without guidance is not enough. Specific team-building skill training is required to be effective and achieve academic success (Cox & Bobrowski, 2004). The described factors can condition research results, as we can see in the following cases.

In an Iranian context, Beigi and Shirmohammadi (2012) observed no significant differences among students with diverse GPA regarding their attitude towards teamwork. Chapman and Van Auken (2001) in North America found a significant but small correlation between student attitudes towards teamwork and GPA. They concluded that students with higher GPA had less positive attitudes towards teamwork. However, in the Spanish context, students’ attitudes towards teamwork based on their previous experiences led to higher academic performance (Martínez-Romero et al., 2021).

Accordingly, although research highlights that teamwork exerts a beneficial influence on academic performance (Lozano-Rodríguez et al., 2020; Park et al., 2015; Martínez-Romero et al., 2021), specific training on developing effective teamwork skills can determine students’ academic success (Cox & Bobrowski, 2004). Considering the results mentioned **above** and the mixed findings for some variables that previous literature has yielded, the following hypotheses are put forward:

H1: There is a relationship between teamwork skills and gender.

H2: There is a relationship between teamwork skills and academic year.

H3: There is a relationship between teamwork skills and GPA.

## Method

This study aims to describe higher education students’ mastery of teamwork skills and its relation to certain socio-academic variables (gender, academic year and grade point average—GPA).

### Participants

With the aim of determining the level of teamwork skills among university students, an observational, transversal descriptive study was designed with an intentional sample of Spanish university students (autonomous community of Galicia). Students are enrolled in social sciences degrees (Education and Business Management). These two degrees have been chosen for two reasons: firstly, they are part of the degrees with the highest number of students in this university, and secondly, their programmes include teamwork as a basic competence to develop.

The Spanish university system includes 82 universities (50 public and 32 private). The total number of students enrolled in 2020-2021 is 1,679,518. Undergraduate students represent 79.8% of enrolled students aged between 18 and 21, and only 5.9% are from other countries, mainly from the EU (2.6%) and Latin America and the Caribbean (1.4%). In the case of universities in the region of Galicia, the percentage of international students is even lower (2.6%). Women represent 56% of the total number of students enrolled (MEC, 2020).

The present sample consists of 615 students from the three public universities of the Region of Galicia. The cultural context of the study is homogeneous, primarily individuals sharing the same cultural background, languages, customs and religion. Male students make up 33% and female students 67%. The average age among participants is 21.52, the minimum being 18 and the maximum 43. The degrees are structured in four years. 31.4% of the students were in their first year, 26% in their second, 25.5% in their third and 17.1% in their final year.

### Measures

#### Socio-academic factors

The proposed questionnaire included a series of variables related to certain socio-academic factors displayed by the participants:

- Gender: this was measured by asking the participants to indicate whether they were men or women.
- Academic year: students were also asked to state which academic year they were in (Spanish degrees are structured in 4 academic years).
- Academic performance: this was measured by asking students to indicate their GPA.

#### Teamwork skills

As stated in the previous literature, there are different tools for measuring teamwork. In our case, we chose the Teamwork Skills Questionnaire (TSQ) (O’Neill et al., 1999), which evaluates the general skill level of an individual in order to participate effectively in teamwork (TSQ) as well as the different components that influence this competence. Despite being a self-reporting tool, it is an excellent way of measuring these cross-curricular skills, given the difficulties in using direct measures (Marshall et al., 2005).

The questionnaire has six sub-scales: (a) adaptability; (b) coordination; (c) decision-making; (d) leadership; (e) interpersonal development; and (f) communication (O’Neil & Mashbun, 1997, 413). This instrument was selected because it measures the most relevant competencies for teamwork.

Adaptability (items: 15, 21, 26, 30, 34) refers to being able to recognise problems at work and respond appropriately. Coordination (items: 6, 11, 17, 23, 32) is understood as an individual’s ability to organise team activities in order to complete a task on time. Decision-making (items: 3, 7, 12, 18, 24, 28) is the ability to use the available information to make team decisions. Leadership (items: 1, 4, 8, 13, 19, 25, 29) refers to the ability to lead a team. Interpersonal development (items: 5, 9, 14, 20, 33, 36) is related to the ability to interact cooperatively with other team members. Communication (items: 2, 10, 16, 22, 27, 31, 35) is the global exchange of clear, precise information.

The Teamwork Skills Questionnaire (TSQ) was chosen because of its reliability rate; the scale has good reliability, offering a range from .84 to .97 (Marshall et al., 2005; O’Neil et al., 2003). The results of our study suggest an adequate level of internal consistency, given that Cronbach’s Alpha ranges from .695 to .868 (Sijtsma, 2009) (Table 1). The psychometric properties of the scale confirm the factor structure of the original questionnaire composed of 36 items and its six factors (*χ*^2^/gl = 3.67, CFI = .937, NNFI = .890, RMSEA = .056) and a very high internal consistency (*α* = .938) (Portela-Pino et al., 2022).

**Table 1:** Cronbach’s alpha coefficients for the Teamwork Skills Scales

Subscale	Alpha
**Adaptability**	.767
**Coordination**	.695
**Decision-making**	.762
**Leadership**	.868
**Interpersonal**	.814
**Communication**	.800
**TSQ**	.939

*N*: 615

### Procedure

The questionnaire was distributed to the students as a form sent through the university platform, thus enabling anonymous, voluntary and confidential participation. Ethical research protocols were respected, emphasising confidentiality and following the ethical rules outlined in the Declaration of Helsinki (AMM, 2017).

### Data analysis

The data analysis procedure has varied depending on the study objective. Descriptive statistics were used to describe the basic features of the data. The next step was to conduct a means analysis using the Student t-test for dichotomous variables and the analysis of variance (ANOVA), followed by the Bonferroni post hoc test for polytomous variables. The effect size was calculated via Cohen’s *d*. In order to establish the relation between the scale variables, Pearson’s correlation was also calculated. Pearson’s chi-square was used to ascertain the association among categorical variables. Finally, a multiple linear regression analysis was carried out to identify the predictors of overall competence on teamwork (TSQ) according to socio-academic variables (method: enter). All the analyses were carried out with a confidence level of 95% through the statistical package SPSS 25.0.

## Results

### Descriptive and univariate analysis

The students’ levels of competence in each of the factors measured are relatively high, except for leadership ($\overline{x}$ = 2.71) and coordination ($\overline{x}$ = 2.99) (Table 2). Thanks to the confidence interval, we can estimate between which values any real population value will fall, with a 5% margin of error, and as we can see, we are very close to the mean.

**Table 2:** Skill level in each of the factors and in total

	M	SD	Min	Max	IC 95%	
					Lower limit	Upper limit
**Adaptability**	3.08	.525	1.00	4.00	3.04	3.12
**Coordination**	2.99	.551	1.00	4.00	2.95	3.03
**Decision-making**	3.06	.517	1.00	4.00	3.01	3.10
**Leadership**	2.71	.638	1.00	4.00	2.66	2.76
**Interpersonal**	3.47	.507	1.00	4.00	3.43	3.51
**Communication**	3.31	.481	1.00	4.00	3.27	3.34
**TSQ**	3.09	.432	1.28	4.00	3.06	3.13

*N*: 615

Hypothesis 1, evidenced in Table 3, which established a relationship between teamwork skills and gender, is accepted. We find that the male students score higher in leadership, whilst female students score higher in adaptability, coordination, interpersonal development and communication. There are no differences in decision-making.

**Table 3:** *T*-student results based on gender for each of the factors, TSQ and GPA

	Gender	***N***	***M***	SD	***t***	Sig.	Cohen’ ***d***
**Adaptability**	Man	203	3.01	.550	− 2.204	.028*	− .188
	Woman	412	3.11	.509			
**Coordination**	Man	203	2.89	.548	− 3.327	.001**	− .283
	Woman	412	3.04	.546			
**Decision-making**	Man	203	3.02	.518	− 1.022	.307	− .874
	Woman	412	3.07	.517			
**Leadership**	Man	203	2.78	.616	2.032	.043*	.174
	Woman	412	2.67	.646			
**Interpersonal**	Man	203	3.34	.548	− 4.522	.000**	− .382
	Woman	412	3.53	.474			
**Communication**	Man	203	3.17	.503	− 4.834	.000**	− .407
	Woman	412	3.37	.456			
**TSQ**	Man	203	3.03	.453	− 2.486	.013*	− .212
	Woman	412	3.12	.418			
**GPA**	Man	203	7.09	1.17	− 3.785	.000**	− .338
	Woman	412	7.45	1.00			

*p < .05,.** *p* < .01; *N*: 615

The effect size was also calculated through Cohen’s *d* (*d* = standardised means difference); the effect is small for all variables except for decision-making, which is large.

Hypothesis 2, evidenced in Table 4, where the academic year is a significant variable in skill level, is partially accepted. It would seem that the further along in their studies a student is, the greater their skill level, especially regarding adaptability and decision-making. Nevertheless, the differences are scarce in the other skills. The effect size was also calculated through Cohen’s *d* (*d* = standardised means difference); the effect is small for variables.

**Table 4:** ANOVA results based on academic year

	Academic year	***N***	***M***	SD	***F***	Sig.	Bonferroni	Cohen’s ***d***
**Adaptability**	**1**	193	2.99	.546	3.542	.014*	First–Fourth = .015*	.017
	**2**	160	3.07	.501				
	**3**	157	3.12	.539				
	**4**	105	3.19	.475				
	**Total**	615	3.08	.525				
**Coordination**	**1**	193	2.92	.555	2.326	.074	No differences	.011
	**2**	160	2.98	.513				
	**3**	157	3.01	.594				
	**4**	105	3.10	.523				
	**Total**	615	2.99	.551				
**Decision-making**	**1**	193	2.97	.549	3.027	.029*	First–second = .045* First–third = .045* First–fourth = .043*	.015
	**2**	160	3.07	.477				
	**3**	157	3.07	.538				
	**4**	105	3.16	.468				
	**Total**	615	3.06	.517				
**Leadership**	**1**	193	2.62	.663	2.338	.073	No differences	.011
	**2**	160	2.71	.603				
	**3**	157	2.80	.662				
	**4**	105	2.74	.594				
	**Total**	615	2.71	.638				
**Interpersonal**	**1**	193	3.43	.537	2.291	.077	No differences	.011
	**2**	160	3.48	.474				
	**3**	157	3.42	.541				
	**4**	105	3.57	.434				
	**Total**	615	3.47	.507				
**Communication**	**1**	193	3.28	.492	1.717	.162	No differences	.008
	**2**	160	3.31	.472				
	**3**	157	3.27	.520				
	**4**	105	3.40	.402				
	**Total**	615	3.31	.481				
**TSQ**	**1**	193	3.04	.445	2.914	.034*	First–Fourth = .024*	.014
	**2**	160	3.10	.400				
	**3**	157	3.11	.479				
	**4**	105	3.19	.362				
	**Total**	615	3.10	.432				

*p < .05; *N*: 615

### Multivariate analysis

Table 5 analyses the correlation between different factors and the independent variable GPA, put forward as hypothesis 3. A positive relation between skills and average academic marks can be found, except for interpersonal development.

**Table 5:** Correlations between each of the factors, overall and GPA

		(1)	(2)	(3)	(4)	(5)	(6)	(7)	(8)
**GPA (1)**	*r*	1							
	Sig.								
**Adaptability (2)**	*r*	.089^*^	1						
	Sig.	.028							
**Coordination (3)**	*r*	.123^**^	.607^**^	1					
	Sig.	.002	0.000						
**Decision-making (4)**	*r*	.085^*^	.729^**^	.708^**^	1				
	Sig.	.036	.000	.000					
**Leadership (5)**	*r*	.095^*^	.567^**^	.594^**^	.645^**^	1			
	Sig.	.018	.000	.000	.000				
**Interpersonal (6)**	*r*	.066	.534^**^	.423^**^	.519^**^	.179^**^	1		
	Sig.	.102	.000	.000	.000	.000			
**Communication (7)**	*r*	.080^*^	.696^**^	.622^**^	.733^**^	.481^**^	.724^**^	1	
	Sig.	.048	.000	.000	.000	.000	.000		
**TSQ (8)**	*r*	.112^**^	.839^**^	.809^**^	.893^**^	.756^**^	.673^**^	.870^**^	1
	Sig.	.006	.000	.000	.000	.000	.000	.000	

*p < .05, **p < .01; *N*: 615

Given that the variables gender, GPA and academic year influence teamwork competence, it is necessary to establish whether there are differences in GPA and academic year for gender. It is observed that women obtain higher GPA than men (see Table 3). Based on these results, we have designed a regression model to explain the socio-academic variables that influence teamwork development according to gender.

Based on the overall objective of this research, a linear multiple regression analysis has been estimated. The results are presented in Table 6. On the one hand, the expected sign for each variable in relation to the dependent variable (overall competence on teamwork—TSQ) is included. On the other hand, both the model’s estimated coefficients (non-standardised) and the typified coefficients (standardised), referred to as *β*, are included. The fourth and fifth columns present the values of the statistic and its significance (*p*-value < .05). The last column shows values VIF, which are lower than 10; this suggests no multicollinearity or internal correlations between the independent variables.

**Table 6:** Multiple linear regression models for the total sample, for men and for women

	Expected sign	Total model					Model for man					Model for woman				
		Model coefficient	***β***	***t***	Sig.	VIF	Model coefficient	***β***	***t***	Sig.	VIF	Model coefficient	***β***	***t***	Sig.	VIF
**Constant**		2.556		19.205	.000**		2.880		13.261	.000**		2.541		15.948	.000**	
**Gender**	+	.075	.082	2.025	.043*	1.026										
**Academic year**	+	.048	.119	2.991	.003**	1.003	.040	.088	1.247	.214	1.010	.050	.133	2.746	.006**	1.001
**GPA**	+	.042	.105	2.596	.010**	1.029	.010	.025	.350	.727	1.010	.064	.152	3.141	.002**	1.001
**Durbin-Watson**		2.081					1.897					2.005				
***R***^**2**^		.034					.008					.040				
***N***		615					203					412				

Dependent variable: overall competence on teamwork (TSQ)

*p < .05; **p < .01

For the total model (men and women), results show a statistically significant relationship between the dependent variable (overall competence on teamwork) and the academic year and GPA of 1% and, with the variable gender, of 5%. All the variables have the expected sign coefficient. Out of this group of variables, the one with the greatest specific weight over the response variable is the academic year (*β* = .119). Accordingly, the regression analysis results show that although the independent variables are significant, their incidence to explain teamwork skills is limited.

In order to find out the academic variables that explain teamwork skills, a model was designed for each gender. Results confirm that both academic variables (GPA and academic year) influence teamwork skills mastery in the case of women, whilst no influence is found in the model for men. Additionally, in the model for women, all the variables have the expected sign coefficient, there is a statistically significant relationship between the dependent variable (overall competence on teamwork) and the academic year and GPA of 1%, and GPA is the variable with the greatest weight with respect to the dependent variable (teamwork) (*β* =.152).

## Discussion, conclusions and implications

University graduates should be efficient in their workplace. For this purpose, not only solid hard skills are required, but competencies that allow them to solve real-life problems. Universities are expected to provide specific training on skills such as problem-solving, critical thinking, cooperation or soft skills (Pöysä-Tarhonen et al., 2016), as the latter would appear to have a close relation with employability. However, training students in these skills is problematic because this type of instruction has not been traditionally contemplated in academic culture (Hirsch, 2017).

For these reasons, having a valid and reliable instrument to measure these skills is of great importance, not only for employers but also for university teaching staff. Results show a strong positive relationship between all the scale factors that vary between .179 and .733, and fundamentally between each of them and the total scale score. In the research carried out by Al-Alawneh et al. (2011), the range varied between .43 and .69, whilst in Brungardt’s study (2009), the correlation varied from .242 to .679. Therefore, this corroborates that the results obtained in our research are similar to previous studies.

### Teamwork skills among students

We can confirm that social science students’ teamwork skills level in each of the soft skills measured is relatively high, except for leadership and coordination. However, it is essential to highlight that some studies have noted that graduates expressed the differences perceived regarding the soft skills acquired at university and those that are actually applied in an organisational context. To this respect, Pereira (2013) confirmed that there were significant differences between students’ perceptions of the soft skills acquired at university and companies’ perceptions. This discrepancy might point to a structural imbalance in the interaction between universities and companies, given that universities do not seem to address the skills demands of the workplace.

### Differences in students’ level of teamwork skills based on socio-academic factors

The results of the study confirm the existence of significant differences in teamwork skills according to gender, academic year and GPA of the students.

#### Teamwork skills and gender

The male students were confirmed to have only obtained higher scores in leadership skills, whereas the female students scored higher in adaptability, coordination, interpersonal development and communication. No differences were noted in decision-making skills.

Our results are in the same line as other studies, such as the one carried out by Al-Alawneh et al. (2011) with Jordan university students. These researchers found significant differences in gender, indicating that women have better interpersonal skills since the mean scores of interpersonal and communication skills were higher for women. According to these authors, this result can be explained by considering Jordan women’s psychology, described to be more serious about their responsibilities and commitments with their families and society in general than Jordan men. It is also worth noting that, although not significant, men obtained higher scores than women in leadership skills. This result is explained by taking into account cultural differences since men are reported to have more opportunities to lead and make decisions than women. Conversely, other studies such as that of Beigi and Shirmohammadi (2012) conducted among students at the University of Iran confirmed a significant relationship but having male students slightly better attitudes towards teamwork than their female counterparts. The results of these two studies should be interpreted considering the cultural context where they occur. It is crucial to consider the influence of gender regarding culture (Galanes et al., 2004) and how it affects teamwork. These differences also could help explain the contradictions found between gender and teamwork performance (Schneid et al., 2015).

Considering the results obtained in our research, we can conclude that there are differences between genders regarding teamwork skills since female students scored higher in all analysed skills, except for leadership. From the point of view of training, these results are highly relevant as teamwork skills are considered fundamental for students’ integration into the job market and their professional development. However, as we have indicated, teamwork skills show significant gender differences.

On the one hand, women seem to show higher self-efficacy in teamwork (Peinado et al., 2015). In this respect, it is worth noting that self-efficacy beliefs influence academic success and students’ motivation to achieve their goals (Saunders et al., 2004; Vera et al., 2011). Equally, several studies confirm that women obtain better academic results than men at university (Khan et al., 2012; Wan Chik et al., 2012). Accordingly, teamwork gender differences seem to affect other highly relevant aspects for academic success, such as GPA.

#### Teamwork skills and academic year

The academic year has also proven to be a significant factor in skills development. It has been shown that the higher the academic year being studied, the higher the level of adaptability and decision-making. This evolution indicates that as students progress through the courses and gain more experience, they develop their skills more efficiently. In addition, First-year students’ reflective capacity, maturity, and commitment may be less developed than in later years (Burdett & Hastie, 2009). Our results align with the study by Rodríguez-Gómez et al. (2018) since they also observed that the students’ teamwork competence level improved as the courses progressed due to students’ perception of more significant development of their acquired skills in the last academic years. As could be expected, this evolution will depend on the specific training methodology employed in different universities and countries. The differences are not as evident as some studies have reported in some cases. For example, in a Jordan context, Al-Alawneh et al. (2011) found no significant differences in the six analysed teamwork skills (coordination, decision-making, leadership, interpersonal development, adaptability, and communication). Nevertheless, these authors also highlight that second-year students obtained higher scores than first-year students, which is consistent with research that recommends teaching generic skills in the second year (Aarnio et al., 2010).

#### Teamwork skills and GPA

Regarding the relationship between GPA and teamwork skills, the results of our research are consistent with other studies. Park et al. (2015), with South Korean university students or Lozano-Rodríguez et al. (2020), with Mexican ones, also found that teamwork skills were positively related to academic performance. In the Spanish context, Martínez-Romero et al. (2021) confirm these findings.

On the other hand, Chamorro-Premuzic et al. (2010), in their study carried out for undergraduate and post-graduate students from UK universities, point out that the scores in these skills are predictors of academic achievement. In this way, developing students’ teamwork skills can transcend all the positive benefits of such decisive competencies and improve their academic scores. Emphasising this connection is highly important, as some education systems neglect teamwork due to the consideration that it interferes with academic achievements (Lau et al., 2014). However, when institutional efforts are made, and specific training on effective teamwork skills is developed, students are more likely to achieve academic success (Cox & Bobrowski, 2004).

As exemplified here, teamwork is not only an essential transferable skill highly valued by employers but a strategic means to obtain better academic results. Students frequently underestimate this connection because they do not usually get grades for teamwork skills (Strom & Strom, 2011). The findings presented are highly relevant as they suggest teamwork can improve not only teamwork skills but also academic performance. Men might need to improve most of their teamwork skills to get higher academic marks and become professionals with more resources. On the other hand, women could consider being open and receptive to lead, trying to change their roles and testing their qualities and strengths. In this way, they could have the chance to transcend any barriers that might limit their capabilities.

Universities provide ideal environments for developing teamwork skills since these skills can be fostered from formal instruction, curriculum design, and non-formal perspectives. From the formal perspective, educational institutions can promote the organisation and implementation of teamwork training programmes. Specifically, teachers can include these skills in the design of their subjects. Some research has emphasised the role of specific innovative teaching techniques in the classroom, such as the micro flip teaching model (Fidalgo-Blanco et al., 2019), Project-based learning (Vogler et al., 2018) or experiential activities (Marasi, 2019).

Likewise, curricular elective subjects and extracurricular training courses have proven to be highly effective for teamwork skills acquisition (Cox & Bobrowski, 2004), so they should be promoted and included at an institutional level. In this way, students can acquire and develop teamwork skills through curricular, academic practices, and university extracurricular or free time leisure activities. Previous literature has observed that extracurricular activities have been shown to positively impact the acquisition of teamwork skills (Sherrod et al., 2002; Zaff et al., 2003). In this sense, Arat (2014) points out that university students also acquire these skills when engaging in activities such as sports, volunteering, art and design projects, long-term workshops and courses, travel, or learning to play an instrument. De Prada et al. (2021) observe that students who participate in musical activities, carry out multidisciplinary experiences in volunteering and participate in international workgroups have better teamwork skills.

Therefore, given the importance of teamwork skills for students’ academic performance and future employability, higher education institutions should endeavour to support and develop teamwork skills training from the first year at university (Burdett & Hastie, 2009; Cox & Bobrowski, 2004; Martínez-Romero et al., 2021) in order to guarantee students’ educational, social and professional success.

## Limitations and future research

Among the limitations of this research, we should indicate that a self-assessment instrument, tested with an intentional sample, was used, so in future research, it would be advisable to carry out a qualitative analysis through interviews or discussion groups to help explain these results. For this reason, results are internally valid, i.e. applicable to the group under study; they cannot be generalised to other groups unless they share the same features. Accordingly, future research should include other degrees to check the differences among the different university training areas. Additionally, the study is based on a specific cultural context, the Spanish one, so replicating this study in other cultural contexts could be helpful to observe the potential effect of culture on teamwork skills.

Lastly, the list of socio-academic factors included in the questionnaire was limited and centred on students’ objective academic experience at university (year of study and GPA). Including new variables and using different analysis models would be interesting to study other factors and activities that might impact teamwork skills acquisition and development. In this sense, variables such as the cultural context, intercultural experiences, students’ field of studies, the presence of specific courses on teamwork training in the curriculum and students’ participation in team-based extra-curricular activities related to sports, music, or volunteering could add valuable insights.

## Abbreviations

GPA: Grade point average
TSQ: Teamwork Skills Questionnaire
VIF: Variance inflation factor
